# A Naturally Occurring Splice Variant of GGA1 Inhibits the Anterograde Post-Golgi Traffic of α_2B_-Adrenergic Receptor

**DOI:** 10.1038/s41598-019-46547-4

**Published:** 2019-07-17

**Authors:** Maoxiang Zhang, Xin Xu, Chunman Li, Wei Huang, Nenggui Xu, Guangyu Wu

**Affiliations:** 1 South China Research Center for Acupuncture and Moxibustion, Guangzhou University of Chinese Medicine, Guangzhou, 510006 China; 2 Department of Pharmacology and Toxicology, Medical College of Georgia, Augusta University, Augusta, GA 30912 USA

**Keywords:** Cell biology, Molecular medicine

## Abstract

The regulatory mechanisms of cell surface targeting of nascent G protein-coupled receptors (GPCRs) *en route* from the endoplasmic reticulum through the Golgi remain poorly understood. We have recently demonstrated that three Golgi-localized, γ-adaptin ear domain homology, ADP ribosylation factor-binding proteins (GGAs) mediate the post-Golgi export of α_2B_-adrenergic receptor (α_2B_-AR), a prototypic GPCR, and directly interact with the receptor. In particular, GGA1 interaction with α_2B_-AR is mediated via its hinge domain. Here we determined the role of a naturally occurring truncated form of GGA1 (GGA1t) which lacks the N-terminal portion of the hinge domain in α_2B_-AR trafficking and elucidated the underlying mechanisms. We demonstrated that both GGA1 and GGA1t were colocalized and mainly expressed at the Golgi. In marked contrast to GGA1, the expression of GGA1t significantly attenuated the cell surface export of newly synthesized α_2B_-AR from the Golgi and in parallel receptor-mediated signaling. Furthermore, we found that GGA1t formed homodimers and heterodimers with GGA1. More interestingly, GGA1t was unable to bind the cargo α_2B_-AR and to recruit clathrin onto the trans-Golgi network. These data provide evidence implicating that the truncated form of GGA1 behaviors as a dominant-negative regulator for the cell surface export of α_2B_-AR and this function of GGA1t is attributed to its abilities to dimerize with its wide type counterpart and to inhibit cargo interaction and clathrin recruitment to form specialized transport vesicles.

## Introduction

G protein-coupled receptors (GPCRs) are the most structurally diverse family of signaling proteins and regulate a variety of cell function. For most GPCRs, the cell surface is the functional destination where they are able to bind to ligands. Agonist binding to GPCRs induces a conformational change of the receptors which in turn activate cognate heterotrimeric G proteins or other signaling molecules, such as arrestins, leading to the activation of a diverse array of downstream effectors. It is apparent that the magnitude and duration of receptor-mediated signaling is, at least in part, controlled by the amount of receptor expression at the cell surface. However, compared to the well-characterized mechanisms underlying the internalization, recycling and degradation pathways^1–3^, how GPCRs transport the cell surface remains poorly defined^4^.

α_2_-Adrenergic receptors (α_2_-ARs) are prototypic GPCRs which play an important role in regulating sympathetic nervous system, both centrally and peripherally. There are three different α_2_-AR subtypes: α_2A_-AR, α_2B_-AR and α_2C_-AR. All three α_2_-ARs have similar structural features, including a relatively large third intracellular loop (ICL3) and a short C-terminus (CT). Numerous studies have demonstrated that, by virtue of their ability to directly interact with many other proteins, both the ICL3 and the CT are the most important domains in the receptors that control almost every function the receptors perform, including G protein-coupling, phosphorylation, signal termination and trafficking^5–7^. Our studies have focused on the elucidation of the molecular mechanisms underlying the intracellular trafficking of α_2_-ARs, particularly their anterograde transport *en route* from the endoplasmic reticulum (ER) through the Golgi to the cell surface^8–17^.

The family of Golgi-localized, γ-adaptin ear domain homology, ADP ribosylation factor (ARF)-binding proteins (GGAs) includes GGA1, GGA2 and GGA3, all of which contain the VHS, GAT, hinge and GAE domains. It has been well defined that all three GGAs function as adaptor proteins for clathrin-coated vesicles derived from trans-Golgi network (TGN) which specifically mediate protein traffic from the TGN to endosomes. One of the important findings in the studies of GGA-mediated trafficking over the past decades is the demonstration that each domain of GGAs is able to interact with other proteins to regulate the trafficking process. In particular, cargo proteins use specific motifs to interact with the VHS domain of GGAs to dictate their sorting to the endosomal transport pathway, whereas specific interaction of GGAs, via the hinge domain, with clathrin is an crucial event required for the recruitment of clathrin onto the TGN, as well as the formation of clathrin-coated vesicles^18–32^. In addition, it has been shown that GGAs may be involved in regulation of the expression of epidermal growth factor receptor and the localization of phosphatidylinositol 4-kinase at the TGN^33,34^. More recently, we have demonstrated that three GGAs regulate α_2B_-AR transport specifically from the Golgi to the cell surface^35,36^.

Each GGA isoform has different spliced variants. However, the precise function of these variants remains unknown. As we have demonstrated that three GGAs use different domains to interact with α_2B_-AR, among them the GGA1 hinge domain is responsible for the interaction with α_2B_-AR^35,36^, in this study we have determined the role of a spliced variant of GGA1 lacking the N-terminal portion of the hinge domain (GGA1t) in the cell surface trafficking of α_2B_-AR. We have demonstrated that this truncated GGA1 variant plays a dominant negative role in the export of α_2B_-AR and its inhibitory function is likely mediated through multiple mechanisms, including dimerization with wild-type counterpart and loss of the abilities to interact with the receptor and to recruit clathrin onto the membrane.

## Results

### Subcellular distribution of GGA1 and GGA1t

**Figure 1:**
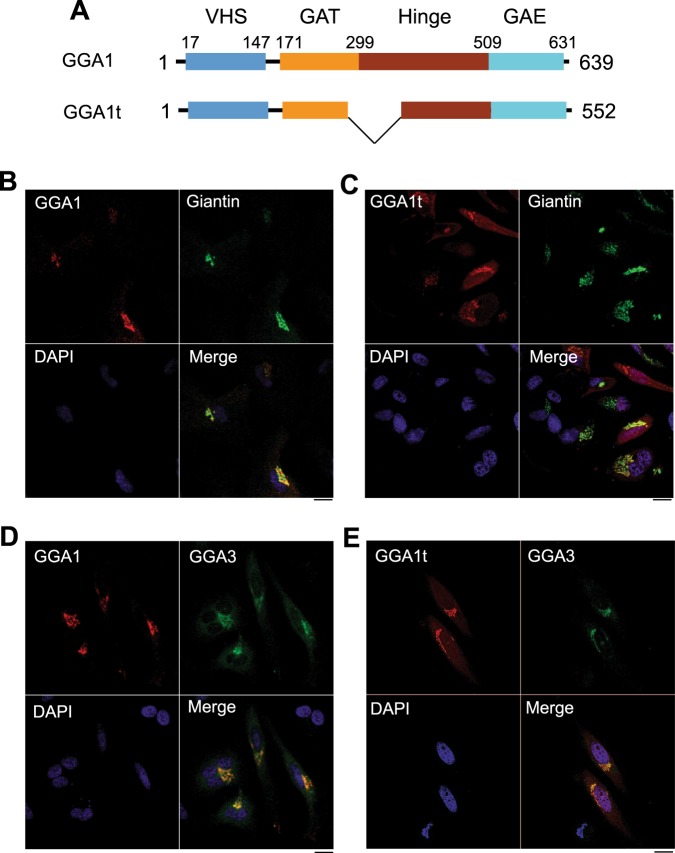
Subcellular localization of GGA1 and GGA1t. (**A**) A diagram showing the domain arrangement of GGA1 and its truncated mutant GGA1t. (**B**,**C**) Colocalization of GGA1 (**B**) and GGA1t (**C**) with the Golgi marker giantin. HeLa cells were transfected with myc-tagged GGA1 (**B**) or GGA1t (**C**) together with venus-tagged giantin and then stained with anti-myc antibodies. The subcellular distribution of GGA1 and GGA1t and their colocalization with giantin were revealed by confocal microscopy. Red, myc-GGA1 (**B**) or myc-GGA1t (**C**); Green, giantin-venus; blue, DNA staining by DAPI. Similar results were obtained in four individual experiments. (**D**,**E**), Colocalization of GGA1 (**D**) and GGA1t (**E**) with GGA3. HeLa cells were transfected with myc-tagged GGA1 (**D**) or GGA1t (**E**) together with GFP-tagged GGA3 and then stained with anti-myc antibodies. Red, myc-GGA1 (**D**) or myc-GGA1t (**E**); Green, GFP-GGA3. Similar results were obtained in three experiments. Scale bars, 10 µm.

GGA1 is composed of 639 amino acid residues. Based on the unigene and uniport databases, there are multiple spliced variants of GGA1. Among these spliced variants, the second form is truncated from L277 to S363 in the GAT and hinge domains and therefore, was designated GGA1t (Fig. 1A). As an initial approach to study the function of GGA1t, we compared the subcellular localization of GGA1 and GGA1t in HeLa cells. Myc-tagged GGA1t and GGA1 were transiently expressed together with venus-tagged giantin, a Golgi marker. Confocal microscopy revealed that both GGA1 and GGA1t were strongly co-localized with giantin (Fig. 1B,C), suggesting their expression on the Golgi apparatus.

We next studied the colocalization of GGA1 and GGA1t with other GGAs. For this purpose, myc-tagged GGA1 or GGA1t was transiently expressed together with green fluorescent protein (GFP)-tagged GGA3 in HeLa cells and their colocalization was visualized by confocal microscopy. Both GGA1 and GGA1t were well colocalized with GFP-GGA3 in HeLa cells (Fig. 1D,E). These data demonstrate that the truncation of the N-terminal portion of the hinge domain does not significantly impact the subcellular distribution of GGA1.

### GGA1t inhibits the cell surface transport from the Golgi and signaling of α_2B_-AR

**Figure 2:**
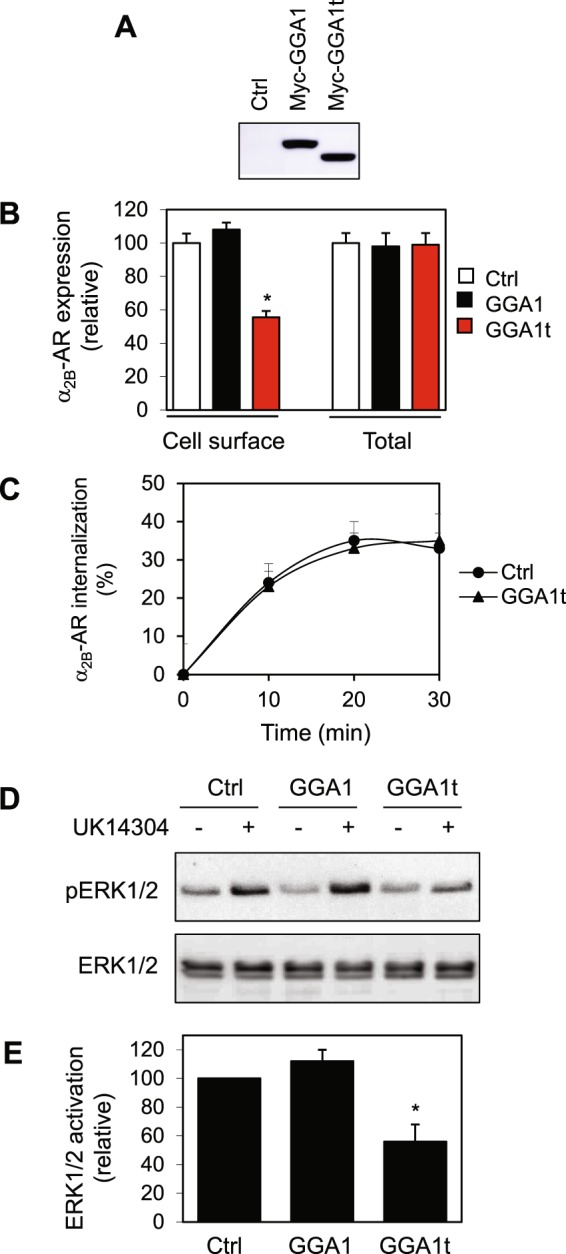
Effects of GGA1 and GGA1t on the cell surface expression of nascent α_2B_-AR. (**A**) A representative blot showing the expression of myc-tagged GGA1 and GGA1t. HEK293 cells were cultured on 6-well dishes and transiently transfected with control vector, myc-GGA1 or myc-GGA1t for 36 h. The expression of GGA1 and GGA1t were determined by immunoblotting using anti-myc antibodies. Similar results were obtained in at least four experiments. (**B**) Effect of GGA1 and GGA1t on the cell surface expression of α_2B_-AR. HEK293 cells inducibly expressing HA-α_2B_-AR were transfected with control vector, GGA1 or GGA1t for 24 h and incubated with doxycycline (40 ng/ml) for 24 h. The cell surface α_2B_-AR expression was determined by intact cell ligand binding using [^3^H]-RX821002 at 20 nM. The data shown are percentages of specific binding obtained from cells transfected with control vector, in which the mean value of specific ligand binding was 38,954 ± 2,786 cpm per well. The total expression of α_2B_-AR was measured by flow cytometry following staining with HA antibodies in permeabilized cells (n = 4). (**C**) Effect of GGA1t on α_2B_-AR internalization. Inducible HEK293 cells expressing α_2B_-AR were transfected with GGA1t and then stimulated with epinephrine at a concentration of 100 μM for 10, 20 and 30 min. The reduction of the cell surface expression of α_2B_-AR was measured by intact cell ligand binding (n = 3). (**D**) Effect of GGA1 and GGA1t on α_2B_-AR-mediated ERK1/2 activation. HEK293 cells inducibly expressing α_2B_-AR were transfected with control vector, GGA1 or GGA1t and incubated with doxycycline at 40 ng/ml for 24 h. After starvation for 3 h, the cells were stimulated with UK14304 at a concentration of 1 µM for 5 min. (**E**) Quantitative data shown in D (n = 3). *p < 0.05 versus control.

We then determined the effect of overexpression of GGA1t on the cell surface expression of α_2B_-AR. In this experiment, α_2B_-AR was tagged with HA at its N-terminus and stably expressed in HEK293 cells using an inducible system. After the cells were transfected with GGA1 or GGA1t and incubated with doxycyclin to induce receptor expression, the numbers of α_2B_-AR expression at the cell surface was quantified by ligand binding of live cells using [^3^H]-RX821002 and the total receptor expression was measured by flow cytometry following staining with high affinity anti-HA antibodies in permeabilized cells. The expression of GGA1t significantly inhibited the cell surface expression of α_2B_-AR as compared with cells transfected with control vector or GGA1 (Fig. 2A,B). In contrast, the expression of GGA1t did not influence overall receptor expression as measured by flow cytometry (Fig. 2B). These data suggest that GGA1t may function as a dominant-negative mutant for the transport of α_2B_-AR to the cell surface.

To determine if GGA1t could influence other trafficking processes of α_2B_-AR, we determined its effect on the internalization of α_2B_-AR. In this experiment, arrestin-3, which was shown to enhance the internalization of α_2B_-AR^37^, was transiently expressed in HEK293 cells that inducibly express α_2B_-AR. The internalization of α_2B_-AR in response to stimulation of epinephrine at each time point was almost identical in control and GGA1t-expressing cells (Fig. 2C), indicating that GGA1t does not affect agonist-induced α_2B_-AR internalization.

α_2B_-AR has been well demonstrated to activate the mitogen-activated protein kinases (MAPK), inhibit adenylyl cyclases and suppress voltage-gated calcium channels. To define if GGA1t overexpression could inhibit the function of α_2B_-AR, the activation of extracellular signal-regulated kinases 1 and 2 (ERK1/2) was used as a functional readout. Consistent with its ability to inhibit α_2B_-AR cell surface transport, transient expression of GGA1t significantly reduced ERK1/2 activation in response to UK14,304 stimulation in cells inducibly expressing α_2B_-AR, as compared to cells transfected with control vector or GGA1 (Fig. 2D,E). These data suggest that GGA1t modulates not only α_2B_-AR cell surface transport but also receptor-mediated signal transduction.

**Figure 3:**
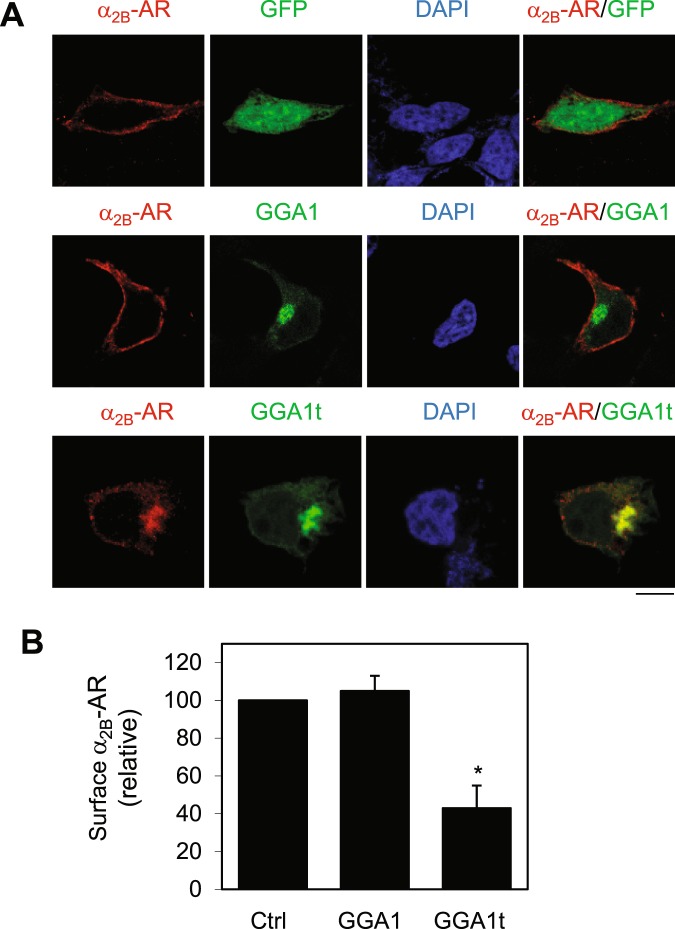
Effect of GGA1 and GGA1t on subcellular localization of α_2B_-AR. (**A**) HEK293 cells inducibly expressing HA-α_2B_-AR were transfected with GFP, GFP-GGA1 or GFP-GGA1t for 36 h and incubated with doxycycline (40 ng/ml) for 24 h. The subcellular distribution of α_2B_-AR was revealed by confocal microscopy following staining with anti-HA antibodies. Red, HA-α_2B_-AR; Green, GFP, GFP-GGA1 or GFP-GGA1; blue, DNA staining of DAPI. Similar results were obtained in at least three experiments. Scale bars, 10 µm. (**B**) Quantitative data of the cell surface expression of α_2B_-AR (n = 45–70 cells).

To further confirm the inhibitory effect of GGA1t on the cell surface transport of α_2B_-AR, GGA1 and GGA1t were tagged with GFP at their N-termini and transiently expressed in HEK293 cells stably expressing HA-α_2B_-AR. The cell surface expression of α_2B_-AR in cells transfected with GGA1t and GGA1 was visualized by confocal microscopy following staining with HA antibodies in permeabilized cells. As expected, α_2B_-AR was robustly expressed at the cell surface in cells expressing GFP alone (Fig. 3A). The expression of GFP-GGA1 did not clearly alter the cell surface expression of α_2B_-AR (Fig. 3A). In contrast, GFP-GGA1t markedly attenuated α_2B_-AR expression at the cell surface and the receptors were partially co-localized with GGA1t (Fig. 3A,B). These data demonstrate that, consistent with the ligand binding data, expression of GGA1t blocks the cell surface export of α_2B_-AR. As GGA1t is mainly expressed at the Golgi (Fig. 1C), these data suggest that it regulates the Golgi-to-cell surface transport of α_2B_-AR.

### Possible homo- and hetero-dimerization of GGA1 and GGA1t

**Figure 4:**
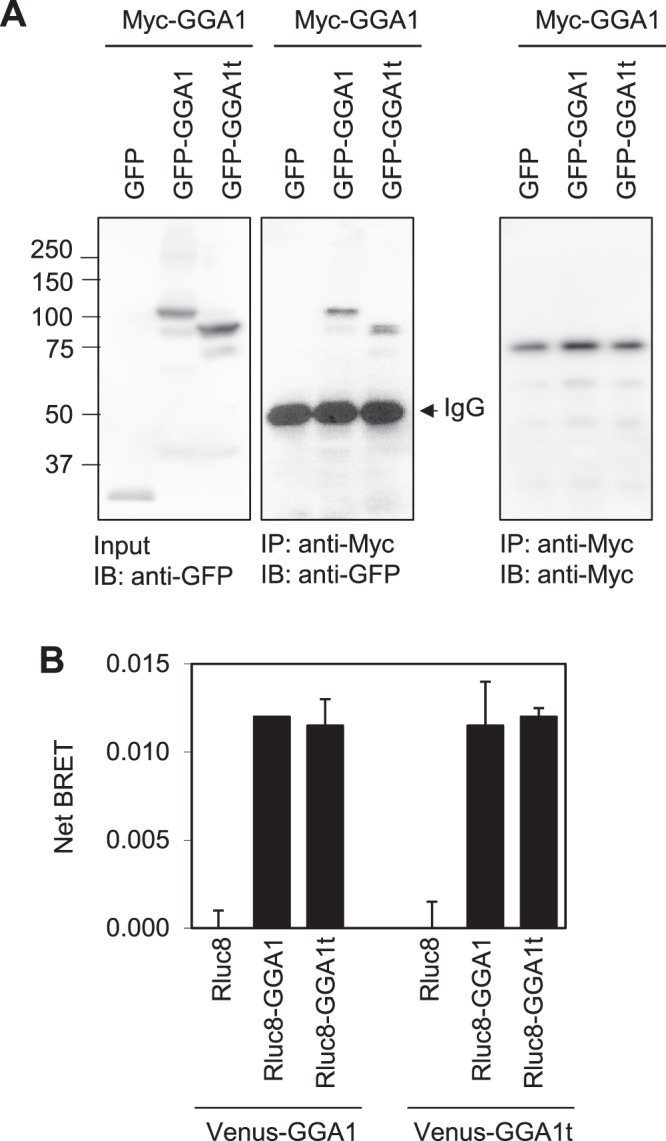
Possible homo- and hetero-dimerization of GGA1 and GGA1t. (**A**) Co-IP of GGA1 and GGA1t. HEK293 cells transfected with myc-GGA1 together with GFP-GGA1 or GFP-GGA1t and immunoprecipitated with anti-myc antibodies. GFP-GGA1 and GFP-GGA1t in the immunoprecipitate were detected by immunoblotting using anti-GFP antibodies. (**B**) Net BRET between GGA1t and GGA1. HEK293 cells were transiently transfected with GGA1 or GGA1t tagged with either venus or Rluc8 and the BRET was measured. The data are presented as the mean ± S.E. of three individual experiments each in triplicate.

We then sought to elucidate the molecular mechanisms underlying the function of GGA1t in regulating α_2B_-AR trafficking. First, we determined if GGA1 and GGA1t were able to form homodimers and heterodimers using co-immunoprecipitation (IP) and bioluminescence resonance energy transfer (BRET) assays. In co-IP assays, HEK293 cells were transfected with myc-GGA1 together with GFP, GFP-GGA1 or GFP-GGA1t, and myc-GGA1 was immunoprecipitated with anti-myc antibodies. The presence of GFP-GGA1 and GFP-GGA1t in anti-myc immunoprecipitates was revealed by immunoblotting using anti-GFP antibodies. Both GFP-GGA1 and GFP-GGA1t, but not GFP alone, were clearly detected in anti-myc immunoprecipitates (Fig. 4A). These data suggest that GGA1 forms a complex with GGA1 and GGA1t.

In BRET assays, GGA1 and GGA1t were tagged with either Rluc8 or Venus and the BRET in live cells was measured after transient expression in HEK293 cells. Consistent with other reports showing GGA1 interacts with itself, a significant BRET was observed in cell transfected with venus-GGA1 and Rluc8-GGA1 whereas no BRET was observed in control cells expressing venus-GGA1 together the empty vector Rluc8 (Fig. 4B). BRET was almost same in cells expressing venus-GGA1t and Rluc8-GGA1t (Fig. 4B). Similar BRETs were observed in cells expressing venus-GGA1 plus Rluc8-GGA1t or venus-GGA1t plus Rluc8-GGA1 (Fig. 4B). These data suggest that GGA1 and GGA1t are able to form homodimers and heterodimers in live cells.

### GGA1t is unable to interact with the cargo α_2B_-AR

**Figure 5:**
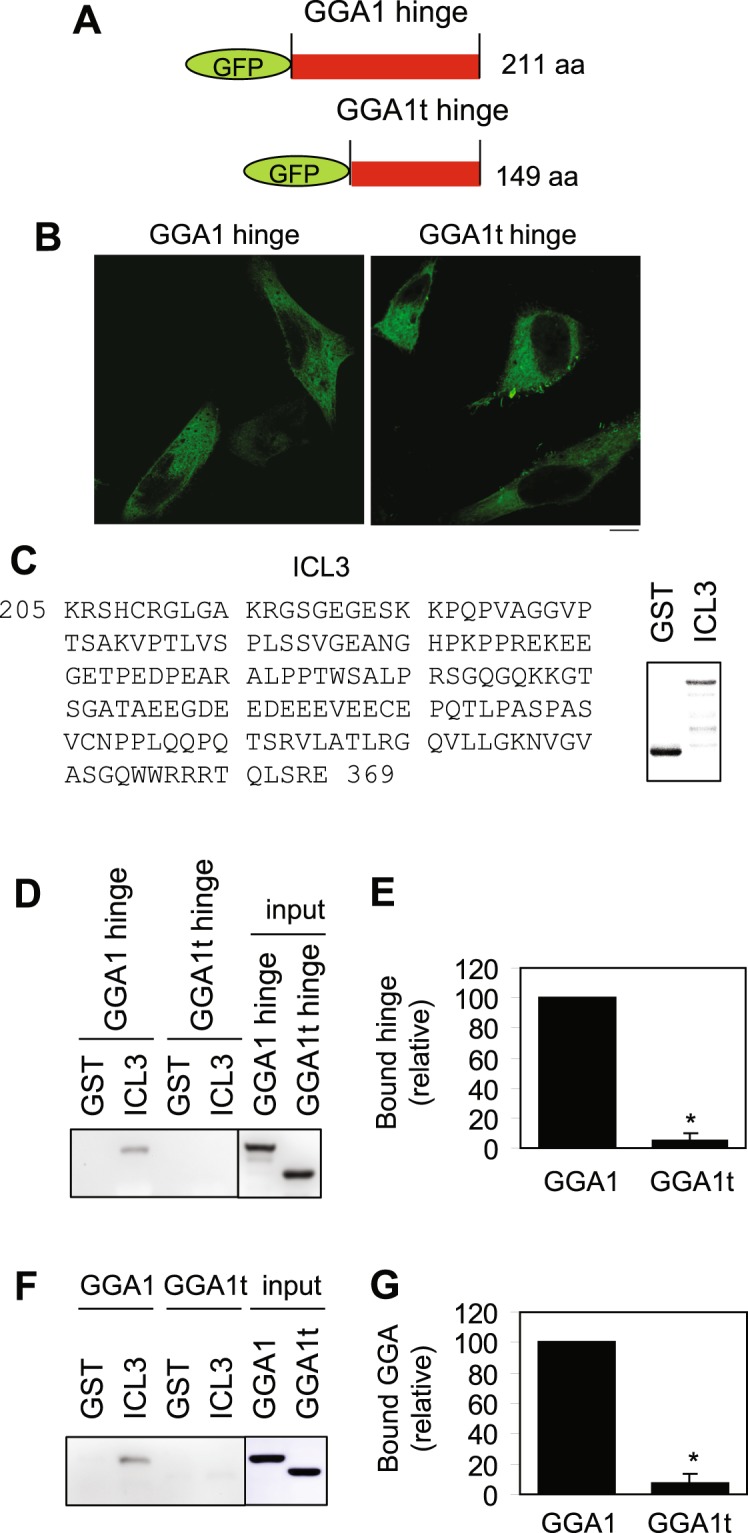
Interaction of GGA1 and GGA1t with the ICL3 of α_2B_-AR in GST fusion protein pulldown assays. (**A**) A diagram showing the generation of GFP-tagged hinge domains of GGA1 and GGA1t. (**B**) Subcellular distribution of GFP-tagged GGA1 and GGA1t hinge domains in HeLa cells. Similar results were obtained in at least three separate experiments. Scale bar, 10 µm. (**C**) The amino acid sequences of the ICL3 of α_2B_-AR (left panel) and Coomassie Brilliant Blue staining of purified GST fusion proteins (right panel). (**D**) Interaction of the α_2B_-AR ICL3 with the hinge domains of GGA1 and GGA1t. The α_2B_-AR ICL3 was generated as GST fusion proteins. The hinge domains of GGA1 and GGA1t were expressed in HEK293 cells and total cell homogenates were incubated with GST-ICL3 fusion proteins. Bound GGA1 and GGA1t were revealed by immunoblotting using anti-GFP antibodies. (**E**) Quantitative data shown in D (n = 3). (**F**) Interaction of the α_2B_-AR ICL3 with full length GGA1 and GGA1t. Myc-tagged GGA1 and GGA1t were expressed in HEK293 cells and total cell homogenates were incubated with GST-ICL3 fusion proteins. Bound GGA1 and GGA1t were revealed by immunoblotting using anti-myc antibodies. (**G**) Quantitative data shown in F (n = 3). Input −5% of total input.

We have recently demonstrated that, although full length α_2B_-AR and GGA1 are not able to form a complex in co-IP assays, the ICL3 of α_2B_-AR and the hinge domain of GGA1 strongly interacted^36^. As the hinge domain is partially deleted in GGA1t, we compared the interaction of α_2B_-AR with GGA1 and GGA1t. In the first experiment, the hinge domains of GGA1 and GGA1t were tagged with GFP (Fig. 5A). Confocal microscopy revealed that both hinge domains were largely expressed in the cytoplasm (Fig. 5B). To measure the interaction of GGA1 and GGA1t hinge domains with the ICL3 of α_2B_-AR, the ICL3 was generated as glutathione S-transferase (GST) fusion proteins (Fig. 5C) and incubated with total cell lysates expressing individual hinge domains. GST-ICL3 fusion proteins, but not GST, strongly bound to the GGA1 hinge domain. In contrast, the GGA1t hinge domain did not bind to the ICL3 (Fig. 5D,E).

In the second experiment, we measured the interactions of full length GGA1 and GGA1t with the ICL3 in GST fusion protein pulldown assays. Consistent with the data observed using the hinge domains, full length GGA1, but not full length GGA1t, interacted with GST-ICL3 (Fig. 5F,G). These data suggest that GGA1t is unable to associate with α_2B_-AR.

### GGA1t is unable to interact with clathrin and recruit clathrin onto the TGN

**Figure 6:**
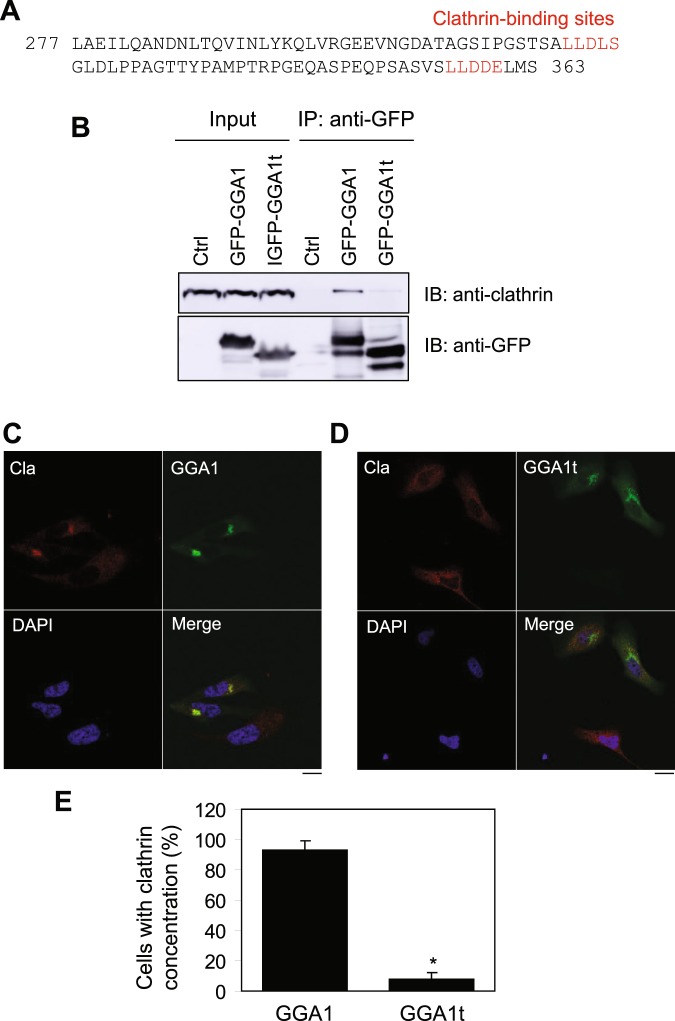
Effect of GGA1 and GGA1t on the recruitment of clathrin onto the TGN. (**A**) Amino acid sequences of GGA1 that are truncated in GGA1t. Two clathrin-binding motifs are shown in red. (**B**) Interaction of GGA1 and GGA1t with clathrin. GFP-tagged GGA1 and GGA1t were transiently expressed in HEK293 cells and immunoprecipitated with anti-GFP antibodies. GGA and clathrin in the immunoprecipitate were detected by immunoblotting using anti-GFP and clathrin antibodies, respectively. In ctrl, cells were not transfected and no antibodies were added. (**C**,**D**) Effect of expression of GGA1 (**C**) and GGA1t (**D**) on the subcellular distribution of clathrin. HeLa cells were transfected with GFP-tagged GGA1 or GGA1t for 36 h. The subcellular distribution of clathrin was revealed by confocal microscopy following staining with clathrin antibodies. Red, clathrin; Green, GFP-tagged GGA1 (**C**) or GGA1t (**D**); blue, DNA staining by DAPI. Scale bars, 10 µm. (**E**) Quantitative data shown in C and D  (n = 5).

GGA1 has been shown to enhance the recruitment of clathrin on the TGN and two clathrin-binding sites have been identified in GGA1 hinge region, As both clathrin-binding sites are deleted in GGA1t (Fig. 6A), we compared the interaction of GGA1 and GGA1t with clathrin in co-IP assays. GGA1 clearly interacted with clathrin, whereas GGA1t did not (Fig. 6B).

We then determined the effect of GGA1 and GGA1t on the recruitment of clathrin on the TGN. Consistent with previous reports^30^, transient expression of GGA1-GFP markedly induced the concentration of clathrin in the perinuclear regions, presumably at the TGN, as compared to cells without transfection of GGA1-GFP (Fig. 6C,E). However, expression of GGA1t-GFP did not affect subcellular localization of clathrin (Fig. 6D,E). These data suggest that, in addition to the loss of its ability to interact with the cargo α_2B_-AR, GGA1t is unable to recruit clathrin onto the Golgi body.

## Discussion

The idea to study the function of truncated GGA1t lacking the N-terminal portion of the hinge domain stems from our recent publications showing that, unlike the interactions between GGAs and other cargoes that bear DxxLL-type motifs and transport to endosomes, three GGAs use different domains to interact with different regions of α_2B_-AR and, in particular, GGA1 interacts with α_2B_-AR via its hinge domain^35,36^.

The most important finding presented here is that GGA1t functions as a dominant-negative regulator in the cell surface export of newly synthesized α_2B_-AR. We have demonstrated that expression of GGA1t markedly inhibited the cell surface expression of α_2B_-AR without altering overall receptor expression. As the experiments were carried out in cells which inducibly express α_2B_-AR, the inhibitory effect is likely due to the reduced export trafficking of newly synthesized receptors. Consistent with the intact cell ligand binding data, subcellular localization analysis by confocal microscopy revealed that α_2B_-AR was indeed expressed in the perinuclear region, presumably the Golgi and TGN compartments, which was in marked contrast to robust cell surface expression in GGA1-expressing cells. Furthermore, expression of GGA1t attenuated α_2_-AR-mediated signaling, which was presumably caused by an attenuation of receptor transport to the cell surface. Altogether, these data strongly demonstrate a dominant negative function of GGA1t in the cell surface transport of α_2B_-AR. However, whether or not GGA1t is a dominant negative regulator in other transport pathways, such as the TGN-endosomes pathway, remains unknown.

There are several possible mechanisms responsible for the dominant-negative effect of GGA1t on the transport of α_2B_-AR. First, co-IP and strong BRET between GGA1 and GGA1t suggest that GGA1t is likely able to form heterodimers with GGA1 in cells. This dimerization may inhibit the normal function of endogenous GGA1 to mediate receptor export to the cell surface.

**Figure 7:**
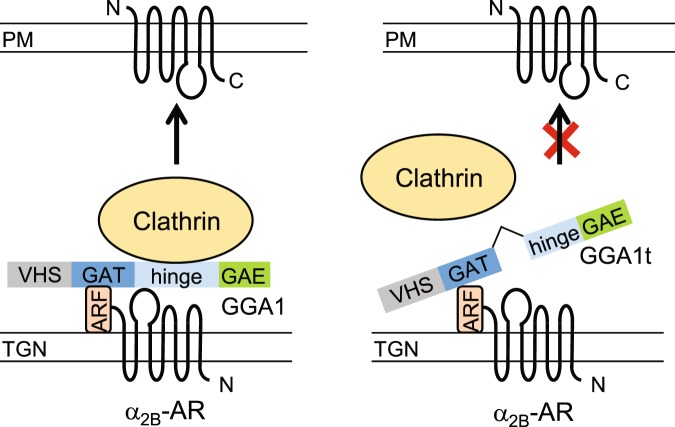
Diagrams showing the possible mechanisms underlying the function of GGA1 and GGA1t in regulating the transport of α_2B_-AR to the plasm membrane (PM). Left panel shows the formation of a unique transport machinery that drives the cell surface transport of α_2B_-AR through specific interactions between α_2B_-AR, ARF1, GGA1 and clathrin. α_2B_-AR interacts with ARF1 and GGA1, via its C-terminus and the ICL3, respectively. The interaction of ARF1 with the GAT domain of GGA1 enhances GGA1 recruitment onto the TGN, whereas the interaction of the hinge domain of GGA1 with clathrin enhances clathrin recruitment. Right panel shows the lack of abilities of GGA1t to interact with α_2B_-AR and clathrin which likely contributes to its dominant-negative effect on the cell surface traffic of the receptor.

Second, GGA1t is unable to interact with the cargo α_2B_-AR. We have shown that GGA1t and its hinge domain were unable to interact with the ICL3 of α_2B_-AR in GST fusion protein pulldown assays. These data identify the α_2B_-AR binding site in the N-terminal portion of the hinge domain. These data further support a crucial role of GGA1-α_2B_-AR interaction in receptor forward trafficking (Fig. 7).

Third, GGA1t is unable to recruit clathrin on the Golgi membrane. The most important function of GGAs is to recruit clathrin on the TGN which is crucial for the formation of clathrin-coated vesicles. There are two clathrin-binding sites identified in the hinge domain which are lost in GGA1t. Consistently, GGA1t did not interact with clathrin and was unable to recruit clathrin on the TGN. Therefore, expression of GGA1t may disrupt the formation of clathrin-coated vesicles (Fig. 7).

Fourth, GGAs are well defined Golgi-localized proteins and their Golgi localization is, at least in part, attributed to the interaction with activated ARF1. The ARF1-binding site has been mapped to the N-terminal portion of the GAT domain which remains intact in GGA1t. Consistently, we have found that GGA1t and GGA1 have very much similar Golgi localization and both are colocalized with GGA3 as revealed by confocal microscopy. It is most likely that GGA1t is able to interact with ARF1, which enhances its localization to the Golgi. In addition, we have previously shown that expression of ARF1 mutants markedly inhibits the cell surface transport of α_2B_-AR and that ARF1 directly interacts with the C-terminus of the receptor which may from a unique transport machinery for the forward trafficking of the receptor (Fig. 7). Although GGA1t interacts with ARF1 and ARF1 interacts with the receptor, these interactions may not be able to form the clathrin-coated vesicles due to the lack of clathrin binding as discussed above. It is reasonable to speculate that GGA1t expression will block the function of endogenous ARF1 which is essential for the transport of the receptor at many different steps, including ER-Golgi and post-Golgi transport^13^.

Physiological significance of spliced GGA variants is not clear at this point. It has been shown that a spliced GGA2 variant containing the N-terminal VHS domain is able to bind to the cytoplasmic domain of sortilin^23^ and that the short isoform of GGA3 is predominantly expressed in different cell lines and tissues^38^. Our data suggest that GGA1t functions as a dominant negative regulator for the cell surface transport of a GPCR. One can image that expression of these dominant negative GGA isoforms will maintain the transport of the receptors at a relatively low level, which can be easily modulated in response to environmental insults by either enhancing the expression of functional GGAs or reducing the expression of their dominant-negative isoforms.

GPCRs represent therapeutic targets of approximately one-third of the drugs on the market to treat human diseases^39^. However, the regulatory mechanisms underlying their targeting to the cell surface, the functional destination for most GPCRs, are largely unexplored. Recent studies have identified a number of specific structural determinants, highly conserved motifs and regulatory proteins (such as Rab GTPases, TBC proteins, ER chaperones, receptor interacting proteins and kinases) to coordinate GPCR export from the ER and the Golgi and transport to the cell surface^8,11,17,40–50^. Identification of GGAs, a family of proteins involved in the transport from the TGN to the endosomal compartment, as well as their naturally occurring mutants to be involved in regulation of the cell surface transport of GPCRs, suggests a novel trafficking function for the GGA family proteins, which may provide an important foundation for the development of novel therapeutic strategies by targeting GPCR anterograde trafficking processes.

## Materials and Methods

### Materials

Antibodies against GFP, myc, clathrin and phospho-specific ERK1/2 were obtained from Santa Cruz Biotechnology, Inc. (Santa Cruz, CA). Anti-ERK antibodies detecting total ERK1/2 expression were from Cell Signaling Technology, Inc. (Beverly, MA). [^3^H]-RX821002 (63 Ci/mmol) was purchased from Perkin Elmer Life Sciences (Waltham. MA). All other materials were obtained as described elsewhere^35,36^.

### Plasmid constructions

α_2B_-AR tagged with three HA (YPYDVPDYA) at its N-terminus in the pcDNA3.1(-) vector was generated as described previously^8^. GGA1t cDNA was cloned from HEK293 cells into the pCMV-Myc vector at the restriction sites of EcoRI and Xhol (forward primer, 5′-GCGAATCCGGGAGCCCGCGATGGAGCCGGAG-3′ and reverse primer, 5′-CCGCTCGAGCTAGAGGCTACCCCAGGTTTC-3′). GFP-tagged GGA1 and GGA3 were generated in the pEGFP-C1 vector as described previously^35,36^. A similar strategy was used to generate GFP-GGA1t. To generate GFP-tagged GGA1 hinge domain (303–513 residues) and GGA1t hinge domain, each domain was generated by PCR and then cloned into the pEGFP-C1 vector. To generate GGA1 and GGA1t tagged with Venus and Rluc8, each was amplified by PCR and then cloned into the Venus-C1 and Rluc8-C1 vectors at the restriction sites of Xhol and EcoRI. The GST fusion protein construct of the third intracellular loop (ICL3, 205–369 residues) of α_2B_-AR was generated using the pGEX-4T-1 vector as described previously^8,14^. The sequence of each construct used in this study was verified by restriction mapping and nucleotide sequence analysis.

### Cell culture and transient transfection

HEK293 and HeLa cells were cultured in Dulbecco’s Modified Eagle’s medium (DMEM) with 10% fetal bovine serum, 100 units/ml penicillin, and 100 µg/ml streptomycin. Transient transfection of cells was carried out using Lipofectamine 2000 reagent (Invitrogen) as described previously^15^.

### Generation of inducible cell lines expressing α_2B_-AR

The Tet-On 3G Tetracycline Inducible Gene Expression System (Clontech Laboratories, Inc.) was utilized to generate stable cell lines inducibly expressing HA-α_2B_-AR in HEK293 cells as described previously^17,35^. Briefly, HA-α_2B_-AR was cloned into the pTRE3G-TRES vector at the BglII and ClaI restriction sites and co-transfected with the PLKO.1 vector in HEK293 cells using Lipofectamine 2000. Inducible α_2B_-AR expression was verified by intact cell ligand binding assays, immunoblotting and confocal microscopy^35^. The current study uses a cell line that expresses 8.5 × 10^5^ receptors per cell.

### Fluorescence microscopy

The subcellular localization of α_2B_-AR, GGAs and clathrin was visualized by fluorescence microscopy as described previously^15^. Briefly, HEK293 cells inducibly expressing HA-α_2B_-AR were transfected with 2 μg of GGA-GFP for 24 h. After induction with doxycycline at 40 ng/ml for 24 h, the cells were permeabilized with buffer containing 0.2% Triton X-100 for 5 min and blocked with 5% normal donkey serum for 1 h. For other co-localization studies, HeLa cells were transiently transfected for 36 h, permeabilized and blocked as described above. The cells were then incubated with antibodies against HA (1:500 dilution), Myc (1:500 dilution) or clathrin (1:100 dilution) for 1 h. After washing, the cells were incubated with Alexa Fluor 594-labeled secondary antibody (1:2000 dilution) for 1 h. The images were captured using a confocal microscope (Zeiss LSM780) and a 63× objective.

### Intact live cell ligand binding

The cell surface expression of α_2B_-AR was measured by ligand binding of intact live cells using [^3^H]-RX821002 as described^8,14^. Briefly, HEK293 cells expressing HA-α_2B_-AR were transiently transfected with control vector, myc-GGA1 or myc-GGA1t for 24 h. The cells were split into 12-well plates and incubated with doxycycline (40 ng/ml) for additional 24 h. The cells were then incubated with DMEM plus [^3^H]-RX821002 (20 nM) in a total volume of 400 μl for 90 min and then washed with ice-cold DMEM to remove excess radioligands. The retained radioligands were extracted by digestion in 1 M NaOH for 2 h. The radioactivity was counted by liquid scintillation spectrometry. For measurement of α_2B_-AR internalization, HEK293 cells expressing α_2B_-AR were cultured on 6-well dishes and transfected with control or GGA1t with 1 μg of arrestin-3 for 24 h. After induction with doxycycline, the cells were starved for 3 h and then stimulated with epinephrine (100 μM) for different time periods. The cells were washed 3 times and the cell surface expression of α_2B_-AR was measured by intact cell ligand binding.

### Flow cytometry

Total α_2B_-AR expression was measured by flow cytometry as described previously^9,51^. Briefly, HEK293 cells expressing HA-α_2B_-AR were suspended in PBS containing 1% fetal calf serum at a density of 4 × 10^6^ cells/ml and permeabilized with 0.2% Triton X-100 in PBS for 5 min on ice. The cells were then incubated with high affinity anti-HA-fluorescein (3F10) at a final concentration of 2 µg/ml at 4 °C for 30 min. After washing with 0.5 ml of PBS twice, the cells were re-suspended and the fluorescence was analyzed on a flow cytometer (Dickinson FACSCalibur).

### Measurement of ERK1/2 activation

Inducible HEK293 cells expressing α_2B_-AR were cultured in 6-well dishes and transfected for 24 h and incubated with tetracycline at 40 ng/ml for 24 h. After starvation for at least 3 h, the cells were then stimulated with UK14304 for 5 min. ERK1/2 activation was determined by immunoblotting using phospho-specific ERK1/2 antibodies as described previously^52^.

### Co-IP assays

HEK293 cells were cultured on 100-mm dishes and transfected with 5 μg of Myc-GGA1 together with 5 μg of pEGFP-C1, GFP-GGA1 or GFP-GGA1t for 48 h. The cells were lysed with 500 μl of lysis buffer (50 mM Tris-HCl, pH 7.4, 140 mM NaCl, 1% Nonidet P-40, and complete Mini protease inhibitor mixture). After rotation for 1 h at 4 °C, the samples were centrifuged at 12,000 g for 15 min. To remove non-specific binding proteins, the cell lysates were incubated with 2 μg of normal mouse IgG and Dynabeads protein G (Life Technologies, Oslo, AS). The supernatants were then incubated with 2 μg of anti-Myc antibodies (Santa Cruz, CA) overnight at 4 °C followed by incubation with 30 μl of Dynabeads protein G for 1 h. The resin was collected and washed four times each with 1 ml of lysis buffer. Immunoprecipitated proteins were eluted with 30 μl of 1x SDS-gel loading buffer and separated by SDS-PAGE. Myc- and GFP-tagged GGA in the immunoprecipitate were detected by immunoblotting using anti-Myc and anti-GFP antibodies, respectively. To study the interaction of GGA1 and GGA1t with endogenous clathrin, HEK293 cells were transfected GFP-GGA1 or GFP-GGA1t and immunoprecipitated with anti-GFP antibodies. GGA and clathrin in the immunoprecipitate were detected by immunoblotting using anti-GFP and anti-clathrin antibodies, respectively.

### BRET assays

Live cell-based BRET assays were carried out as described previously^14,53,54^. HEK293 cells were cultured on 6-well dishes and transfected with 0.1 μg of GGA-Rluc8 and 1.5 μg of GGA-Venus for 24 h. The cells were transferred to black 96-well plates. Coelenterazine h (5 mM) was added to all wells immediately prior to making measurements. Raw BRET signals were calculated by dividing the emission intensity at 520–545 nm by the emission intensity at 475–495 nm. Net BRET was this ratio minus the same ratio measured from cells expressing only the BRET donor (Rluc8).

### GST fusion protein pulldown assays

GST fusion proteins were purified as described previously^8,14^. Purified GST fusion proteins were used immediately or stored at 4 °C for no longer than 3 days. GST fusion proteins tethered to the glutathione resin were incubated with total cell lysates in 500 µl of buffer (20 mM Tris-HCl, pH 7.5, 1% NP-40, 140 mM NaCl and 1 mM MgCl_2_) at 4 °C for 4–6 h. The resin was washed 4 times with 0.5 ml of binding buffer and the retained proteins were solubilized in SDS-gel loading buffer and separated by SDS-PAGE. Proteins bound to GST fusion proteins were detected by immunoblotting.

### Statistical analysis

Differences were evaluated using Student’s *t* test, and *p* < 0.05 was considered as statistically significant. Data are expressed as the mean ± S.E.

## Supplementary information

Supplementary info
