# Supplementary material for: A Naturally Occurring Splice Variant of GGA1 Inhibits the Anterograde Post-Golgi Traffic of α2B-Adrenergic Receptor
Source: Sci Rep. 2019 Jul 17;9:10378. doi: 10.1038/s41598-019-46547-4 (PMC6637153; doi:10.1038/s41598-019-46547-4)

Supplementary Information

A Naturally Occurring Splice Variant of GGA1 Inhibits the Anterograde Post-Golgi Traffic of α_{2B}-Adrenergic Receptor

Maoxiang Zhang[1,2], Xin Xu[2], Chunman Li[2], Wei Huang[2], Nenggui Xu[1] and Guangyu Wu[2]*

[1]South China Research Center for Acupuncture and Moxibustion, Guangzhou University of Chinese Medicine, Guangzhou, 510006, China

[2]Department of Pharmacology and Toxicology, Medical College of Georgia, Augusta University, Augusta, GA 30912, USA

Supplementary Figure 1S. Full Western blot images shown in the manuscript

Figure 2D

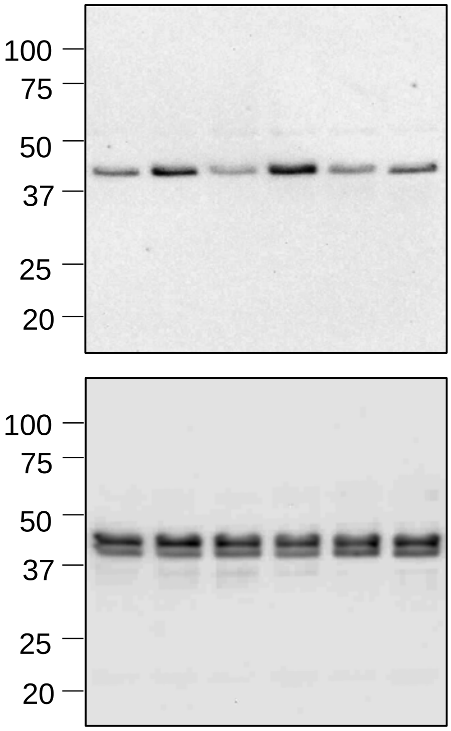

Figure 2A

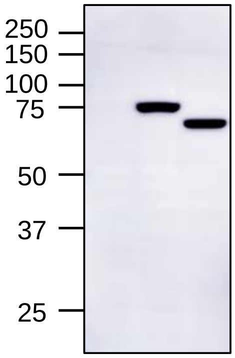

Figure 5D

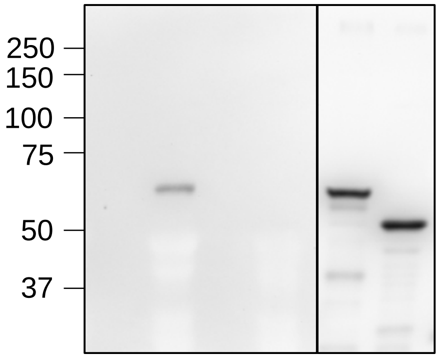

Figure 6B

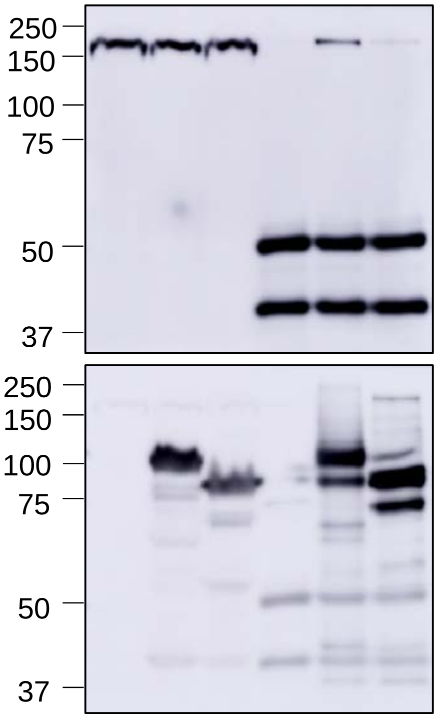

Figure 5F

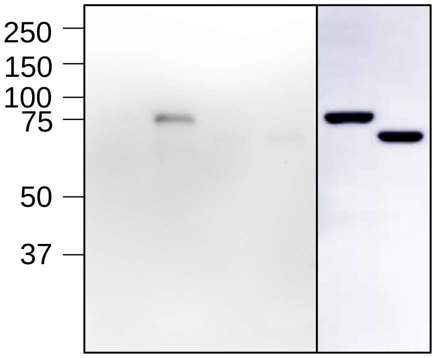

Supplement: Supplementary file 1 — Supplementary info [file 41598_2019_46547_MOESM1_ESM.pdf]
